# Prognostic and clinicopathological significance of MLKL expression in cancer patients: a meta-analysis

**DOI:** 10.1186/s12885-018-4655-4

**Published:** 2018-07-13

**Authors:** Binwu Hu, Deyao Shi, Xiao Lv, Songfeng Chen, Qin Huang, Mao Xie, Zengwu Shao

**Affiliations:** 1 Department of Orthopaedics, Union Hospital, Tongji Medical College, Huazhong University of Science and Technology, Wuhan, 430022 China; 2 Department of Orthopaedic Surgery, The First Affiliated Hospital of Zhengzhou University, Zhengzhou, China; 3 Department of Medical Rehabilitation, Union Hospital, Tongji Medical College, Huazhong University of Science and Technology, Wuhan, 430022 China

**Keywords:** MLKL, Necroptosis, Cancer, Prognosis, Meta-analysis

## Abstract

**Background:**

MLKL is the most important executor of necroptosis pathway. Recent studies have demonstrated that MLKL could serve as a potential prognostic biomarker for cancer patients. However, most studies reported so far are limited in discrete outcome and sample size.

**Methods:**

We systematically searched PubMed, Embase, Web of Science and CNKI to obtain all relevant articles about the prognostic value of abnormally expressed MLKL in patients with any type of tumor. Odds ratios or hazards ratios (HRs) with corresponding 95% confidence intervals (CIs) were pooled to estimate the association between MLKL expression and clinicopathological characteristics or survival of cancer patients.

**Results:**

A total of 6 eligible studies with 613 cancer patients were enrolled in our meta-analysis. Our results demonstrated that decreased expression level of MLKL was significantly associated with poor overall survival (OS) (pooled HR 0.26, 95%CI 0.17–0.40, high/low) and event-free survival (EFS) (pooled HR 0.45, 95%CI 0.23–0.87, high/low) in cancer patients. Furthermore, subgroup analysis divided by type of cancer, sample size, follow-up time and Newcastle–Ottawa Scale (NOS) score showed consistent prognostic value. In addition, our analysis revealed that decreased expression level of MLKL was significantly associated with advanced tumor stage, more lymph node metastasis and older age.

**Conclusions:**

In conclusion, our meta-analysis suggested that decreased MLKL expression might be a convinced unfavorable prognostic factor that could help the clinical decision-making process.

## Background

Despite the great development of therapies over past years, cancer is still the major public health problem worldwide [1]. Innumerable new cancer cases and cancer deaths are predicted to occur in world every year [2]. Considering the poor prognosis of most cancers, numerous researchers have devoted to identifying novel cancer biomarkers, not only for predicting prognosis but also for targeted therapy [3]. However, most cancer biomarkers identified so far are not satisfactory [4]. Therefore, it is urgently needed to discover novel biomarkers for cancer.

Necrosis, which is regarded as a kind of unprogrammed cell death previously, is characterized by plasma membrane permeabilization, cellular collapse, swelling of the organelles, mitochondria dysfunction and inflammation in the surrounding tissues [5]. Necrosis is a common feature of solid tumors and many cancer treatments including chemotherapy and radiation could induce necrosis of tumor cells [6, 7]. However, accumulating evidence has revealed that necrosis could occur in a controlled and regulated manner, which is referred to as necroptosis [8]. Necroptosis is a caspase-independent form of regulated cell death, which is executed by receptor-interacting protein kinase 1 (RIPK1)–receptor-interacting protein kinase 3 (RIPK3) –mixed lineage kinase domain-like protein (MLKL) [9]. More specifically, RIPK1 kinase activity leads to the phosphorylation of RIPK3, and subsequently, RIPK3 phosphorylation further causes MLKL phosphorylation and trimerization. MLKL homotrimer then translocates to the plasma membrane and causes necrotic plasma membrane permeabilization, which is one of the necroptosis executing mechanisms [10]. An increasing number of studies have demonstrated that downregulation or mutations of necroptosis regulators including RIPK1, RIPK3, MLKL and CYLD are frequently found in human tumors, which indicates the pivotal roles of necroptosis in the pathogenesis of cancer [11–15]. It has been reported that loss of RIPK1 was strongly associated with metastatic disease in head and neck squamous cell carcinoma patients [16]. In non-Hodgkin lymphoma, polymorphisms in the RIPK3 gene were identified and found to be associated with increased risk of tumors [17]. Koo et al. showed that RIPK3 expression was reduced in most of breast cancer patients, suggesting that RIPK3 deficiency was positively selected during tumor growth/development [13]. It is widely accepted that evasion of cell death is one of the hallmarks of cancer [18]. Resistance to apoptosis, the most widely explored form of programmed cell death, has been proven to be related to the poor prognosis of cancer patients [19].Therefore, triggering necroptosis might be an alternative way to eradicate cancer cells especially for apoptosis-resistant cancer cells. Recently, necroptosis-based cancer therapy has been proposed to be a novel strategy for antitumor treatment. It has been proven that necroptosis inducers could trigger necroptosis in plenty of cancer cell lines [6]. Thus, we could speculate that the sensitivity of cancer patients to necroptosis inducers might be related to their prognosis. However, the prognostic value of necroptosis has not been fully elucidated before.

MLKL is the most important executor of necroptosis pathway. Plasma membrane translocation of trimerized MLKL protein is essential for necroptosis, leading to permeabilization of endoplasmic reticulum, mitochondria and lysosome [20]. MLKL also plays pivotal roles in the pathogenesis of cancer. Abnormal expression of MLKL has been detected in many kinds of tumors, such as breast cancer, colon cancer, ovarian cancer and gastric cancer [21–24]. Recent studies have revealed that MLKL could serve as a potential prognostic biomarker for patients with cancer [21–23, 25, 26]. However, most studies reported so far are limited in discrete outcome and sample size. Therefore, we performed the current meta-analysis to elucidate the prognostic and clinicopathological significance of MLKL expression in patients with cancer.

## Methods

### Study strategy

The present review was performed in accordance with the standard guidelines for meta-analysis and systematic reviews of tumor marker prognostic studies [27, 28]. The database PubMed, Embase, Web of Science and CNKI were independently searched by two researchers (Binwu Hu and Deyao Shi) to obtain all relevant articles about the prognostic value of abnormally expressed MLKL in patients with any tumor. The literature search ended on January 20, 2018. The search strategy used both MeSH terminology and free-text words to increase the sensitivity of the search. The search strategy was: “MLKL or mixed lineage kinase domain-like protein” AND “cancer or tumor or carcinoma or neoplasm or malignancy”. We also screened the references of retrieved relevant articles to identify potentially eligible literatures. Conflicts were solved through group discussion.

### Inclusion and exclusion criteria

Studies included in this analysis had to meet the following inclusion criteria: (1) Patients were pathologically diagnosed with any type of cancer; (2) MLKL expression was determined in human tissues or plasma samples using any techniques; (3) Patients were divided into high and low expression or positive and negative expression groups, the relationship between MLKL expression levels with survival outcome was investigated; (4) Sufficient published data or the survival curves were provided to calculate hazard ratios (HR) for survival rates and their 95% confidence intervals (CI). Exclusion criteria were as follow: studies without usable or sufficient data, studies using non-human samples, reviews, laboratory articles, case reports, letters, unpublished articles and conference abstracts. All eligible studies were carefully screened by two researchers (Binwu Hu and Deyao Shi), and discrepancies were resolved by discussing with a third researcher (Xiao Lv).

### Data extraction

Two investigators (Binwu Hu and Deyao Shi) extracted relevant data independently and reached a consensus on all items. For all eligible studies, the following information of each article was collected: author, year of publication, tumor type, expression associated with poor prognosis, Newcastle–Ottawa Scale (NOS) score, method of obtaining HRs and the characteristics of the study population (including country of the population enrolled, number of patients (high/low), follow up (month)), endpoints, assay method, cut-off value and survival analysis. For endpoints, overall survival (OS), disease-free survival (DFS) and recurrence -free survival (RFS) were all regarded as endpoints. In addition, DFS and RFS were redefined as event-free survival (EFS) in our article. We employed HR which was extracted following a methodology suggested previously to evaluate the influence of MLKL expression on prognosis of patients [29]. If possible, we also asked for original data directly from the authors of the relevant studies.

### Quality assessment

Quality of all included studies was assessed independently by three researchers (Songfeng Chen, Qin Huang and Mao Xie) using the validated NOS, and disagreements were resolved through discussion with another researcher (Xiao Lv). This scale uses a star system to evaluate a study in three domains: selection of participants, comparability of study groups, and the ascertainment of outcomes of interest. We considered studies with scores more than 6 as high-quality studies, and those with scores no more than 6 as low-quality studies.

### Statistical analysis

Statistical analysis was performed using Stata Software 14.0 (Stata, College Station, TX). Pooled HRs (high/low) and their associated 95% CIs were used to analyze the prognostic role of MLKL expression in various cancers. Pooled odds ratios (ORs) (low/high) and their associated 95% CIs were used to analyze the association between MLKL expression level with clinicopathological parameters. The heterogeneity among studies was evaluated using Cochran’s Q and I^2^ statistics. A *p* value less than 0.10 or an I^2^ value larger than 50% were considered statistically significant. The fixed-effect model was used for analysis without significant heterogeneity between studies (*p* > 0.10, I^2^ < 50%). Otherwise, the random-effect model was chosen. To explore the source of heterogeneity, subgroup analysis was preformed through classifying the included studies into subgroups according to similar features. We also conducted sensitivity analysis to test the effect of each study on the overall pooled results. In addition, for the studies from which we could obtain clinicopathological characteristics, we calculated the pooled ORs and performed heterogeneity tests to analyze the relationship between MLKL expression level with lymph node metastasis, tumor stages, age, differentiation grade and gender in different types of cancers. Due to the limited number of studies (below 10) included in this analysis, publication bias was not assessed.

## Results

### Characteristics of studies

**Fig. 1:**
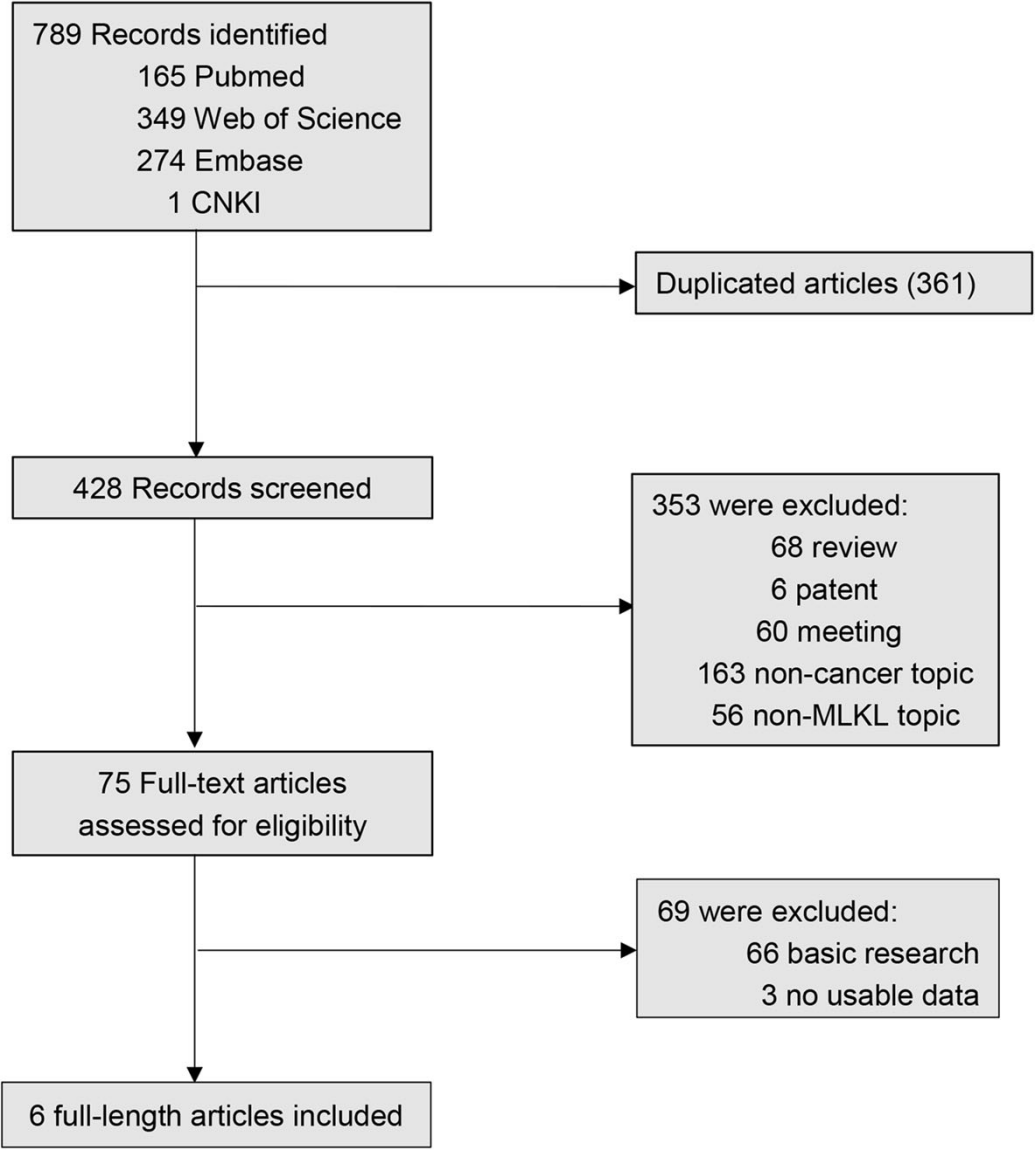
The flow diagram indicated the process of study selection

According to our search strategy, a total of 789 studies were retrieved. Among these researches, the following studies were excluded: duplicate (*n* = 361), review (*n* = 68), patent (n = 6), meeting abstract (*n* = 60), studies describing non-cancer topic (*n* = 163), studies describing non-MLKL topic (*n* = 56), studies belonging to basic research (*n* = 66) and studies lacking relevant data (n = 3). Eventually, 6 studies meeting the inclusion criteria were included in this meta-analysis. The screening process and results are shown in Fig. 1, and the main characteristics of the included studies are shown in Table 1**.** Among these studies, a total of 613 patients were included, with a maximum sample size of 153 patients and a minimum sample size of 54 patients (Mean 102.0). The accrual period of these studies ranged from 2013 to 2017. The regions represented in this study included the China (5) and the United States of America (1). Six different types of cancer were evaluated including breast cancer, gastric cancer, cervical squamous cell carcinoma, ovarian cancer, colon cancer and pancreatic adenocarcinoma. Among these studies, OS, DFS and RFS were estimated as survival outcome in 100% (6/6), 17% (1/6) and 33% (2/6) of the studies respectively. DFS and RFS were combined together into EFS, which was regarded as prognostic parameter in our study. To evaluate the expression of MLKL, all studies used immunohistochemistry (IHC) technique. Because the cut-off definitions were various, the cut-off values were different in these studies.

**Table 1:** Characteristics of studies included in the meta-analysis

Author	Year	Region	Type of Cancer	Sample size (high/low)	Follow-up (month)	Endpoints	Expression associated with poor prognosis	Assay method	Cut-off value	Survival analysis	NOS score	Method
Zhu et al.	2017	China	Breast Cancer	27/47(74)	94	OS	Low	IHC	High: the sum of the staining intensity and extent scores was higher than 3	NA	6	2
Zhai et al.	2016	China	Gastric Caner	50/67(117)	114	OS	Low	IHC	High: IHC score was no less than 2	Univariate Multivariate	7	2
Ruan et al.	2015	China	Cervical Squamous Cell Carcinoma	25/29(54)	80	OS	Low	IHC	High: the sum of the staining intensity and extent scores was no less than 4	NA	6	2
He et al.	2013	China	Ovarian Cancer	75/78(153)	102	OS,DFS	Low	IHC	High: the sum of the staining intensity and extent scores was no less than 4	Univariate Multivariate	7	1
Li et al.	2016	China	Colon Cancer	83/52(135)	95.3	OS,RFS	Low	IHC	High: the sum of the staining intensity and extent scores was no less than 4	Univariate Multivariate	7	1
Colbert et al.	2013	USA	Pancreatic Adenocarcinoma	74/6(80)	114	OS,RFS	Low	IHC	High: the sum of the staining intensity and extent scores was higher than 1	Univariate Multivariate	7	1

*IHC* immunohistochemistry, *NA* not available, *NOS* Newcastle-Ottawa Scale, Method: 1 denoted as obtaining HRs directly from publications; 2 denoted as HRs calculated from the total number of events, corresponding p value and data from Kaplan-Meier curves

### Association between MLKL expression levels with OS of cancer patients

**Fig. 2:**
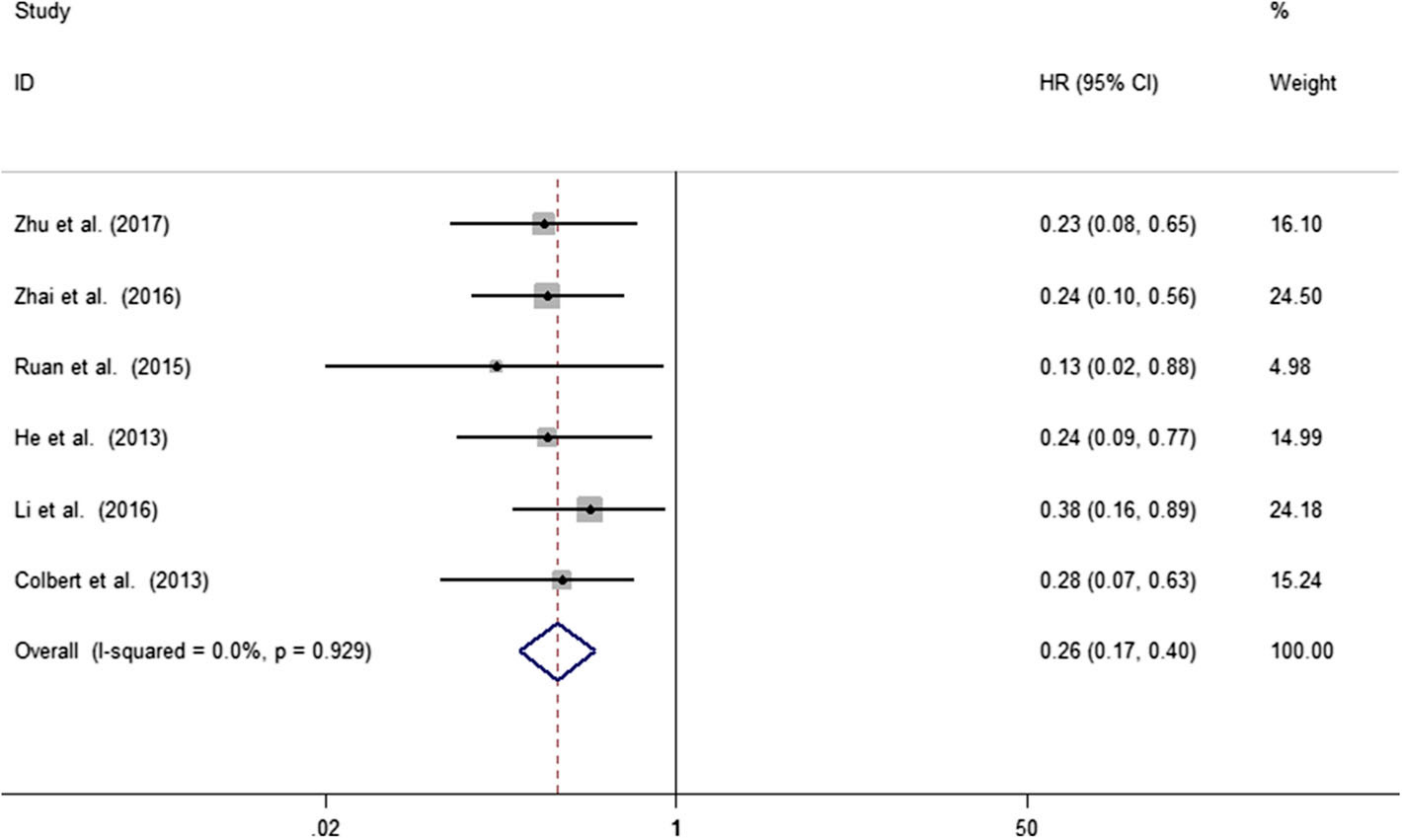
Meta-analysis of the pooled HRs of OS for cancer patients

**Fig. 3:**
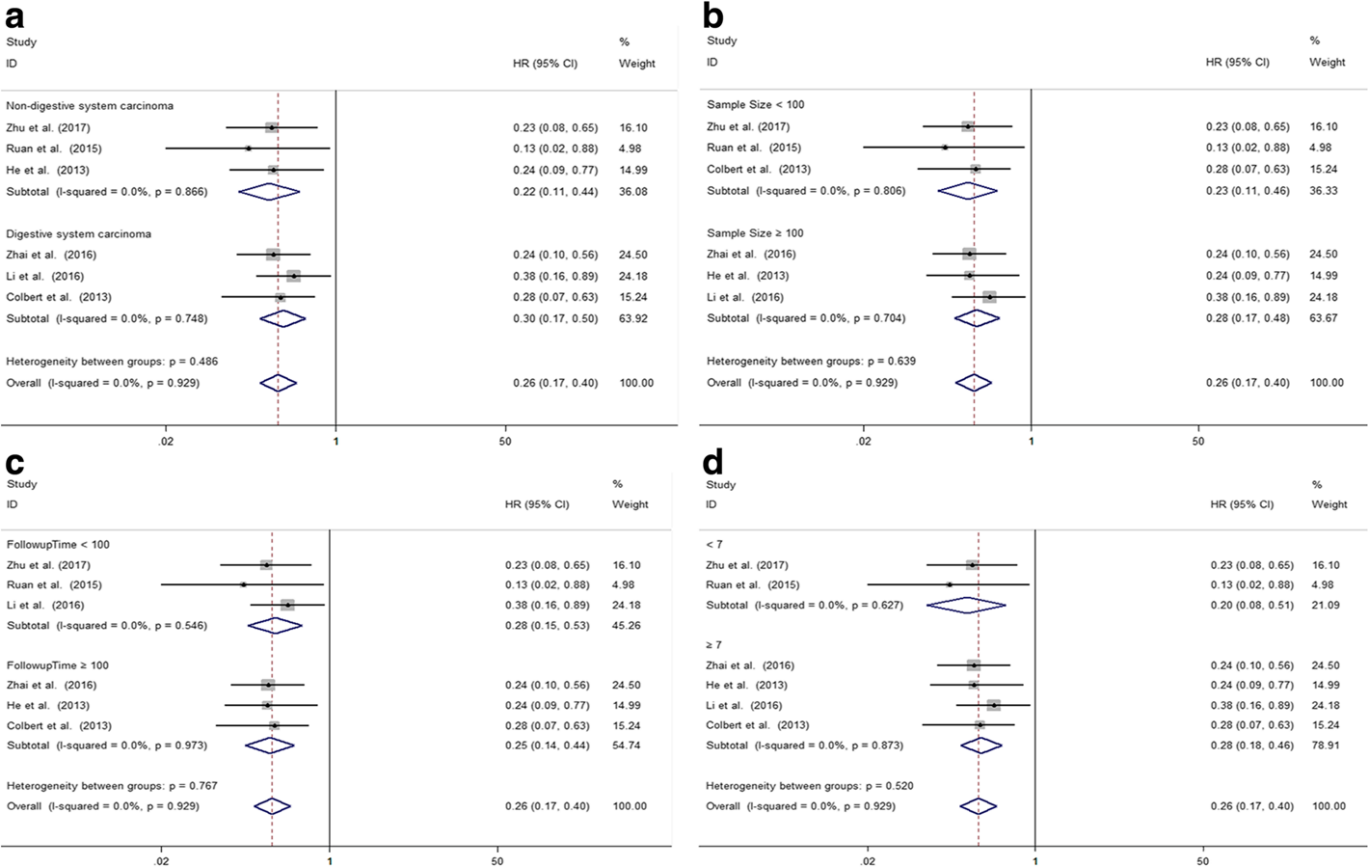
Subgroup analysis of pooled HRs for OS in cancer patients. **a** Subgroup analysis stratified by type of cancer. **b** Subgroup analysis stratified by sample size. **c** Subgroup analysis stratified by follow-up time. **d** Subgroup analysis stratified by NOS score

A total of 6 studies involving 613 patients reported the relationship between abnormal expression levels of MLKL with OS of cancer patients. We used fixed-effect model to calculate the pooled HR. The pooled HR for OS was 0.26 (95%CI: 0.17–0.40, *p* < 0.001), which indicated that decreased expression level of MLKL was significantly associated with poor OS in cancer patients (Fig. 2). The test for heterogeneity showed no significant results (X^2^ = 1.35, *p* = 0.929; I^2^ = 0%). In order to further explore the association between abnormal expression levels of MLKL with OS in cancer patients, subgroup analysis was performed based on the following factors: type of cancer (non-digestive system or digestive system malignancies), sample size (fewer than 100 or more than 100), follow-up time (fewer than 100 or more than 100 months), paper quality (NOS scores < 7 or ≥ 7). The subgroup analysis illustrated the same results that the significant association between decreased expression levels of MLKL with poor OS of cancer patients was not altered with all the factors above (Table 2). For type of cancer, non-digestive system malignancies (HR = 0.22, 95%CI: 0.11–0.44, *p* < 0.001) and digestive system malignancies (HR = 0.30, 95%CI: 0.17–0.50, p < 0.001) (Fig. 3a). For sample size, fewer than 100 (HR = 0.23, 95%CI: 0.11–0.46, p < 0.001) and more than 100 (HR = 0.28, 95%CI: 0.17–0.48, *p* < 0.001) (Fig. 3b). For follow-up time, fewer than 100 months (HR = 0.28, 95%CI: 0.15–0.53, *p* < 0.001) and more than 100 months (HR = 0.25, 95%CI: 0.14–0.44, p < 0.001) (Fig. 3c). For paper quality, NOS scores < 7 (HR = 0.20, 95%CI: 0.08–0.51, *p* = 0.001) and NOS scores ≥7 (HR = 0.28, 95%CI: 0.18–0.46, *p* < 0.001) (Fig. 3d). No significant heterogeneity was found across studies in all the subgroup analysis (Table 2).

**Table 2:** Subgroup analysis of pooled HRs for OS in cancer patients with abnormally expressed MLKL

Subgroup analysis	No. of studies	Pooled HRs	Heterogeneity	
		Fixed	I^2^	*p* -value
Type of cancer				
Non-digestive system carcinoma	3	0.22[0.11–0.44]	0%	0.866
Digestive system carcinoma	3	0.30[0.17–0.50]	0%	0.748
Sample size				
< 100	3	0.23[0.11–0.46]	0%	0.806
≥ 100	3	0.28[0.17–0.48]	0%	0.704
Follow-up time				
< 100	3	0.28[0.15–0.53]	0%	0.546
≥ 100	3	0.25[0.14–0.44]	0%	0.973
NOS score				
< 7	2	0.20[0.08–0.51]	0%	0.627
≥ 7	4	0.28[0.18–0.46]	0%	0.873

Using HRs of Cox multivariate analysis from three studies, we found that decreased MLKL expression was an independent prognostic factor for OS in cancer patients (HR = 0.31, 95%CI: 0.17–0.54, p < 0.001). Furthermore, there was no significant heterogeneity among studies (*p* = 0.787; I^2^ = 0%).

### Association between MLKL expression levels with EFS of cancer patients

**Fig. 4:**
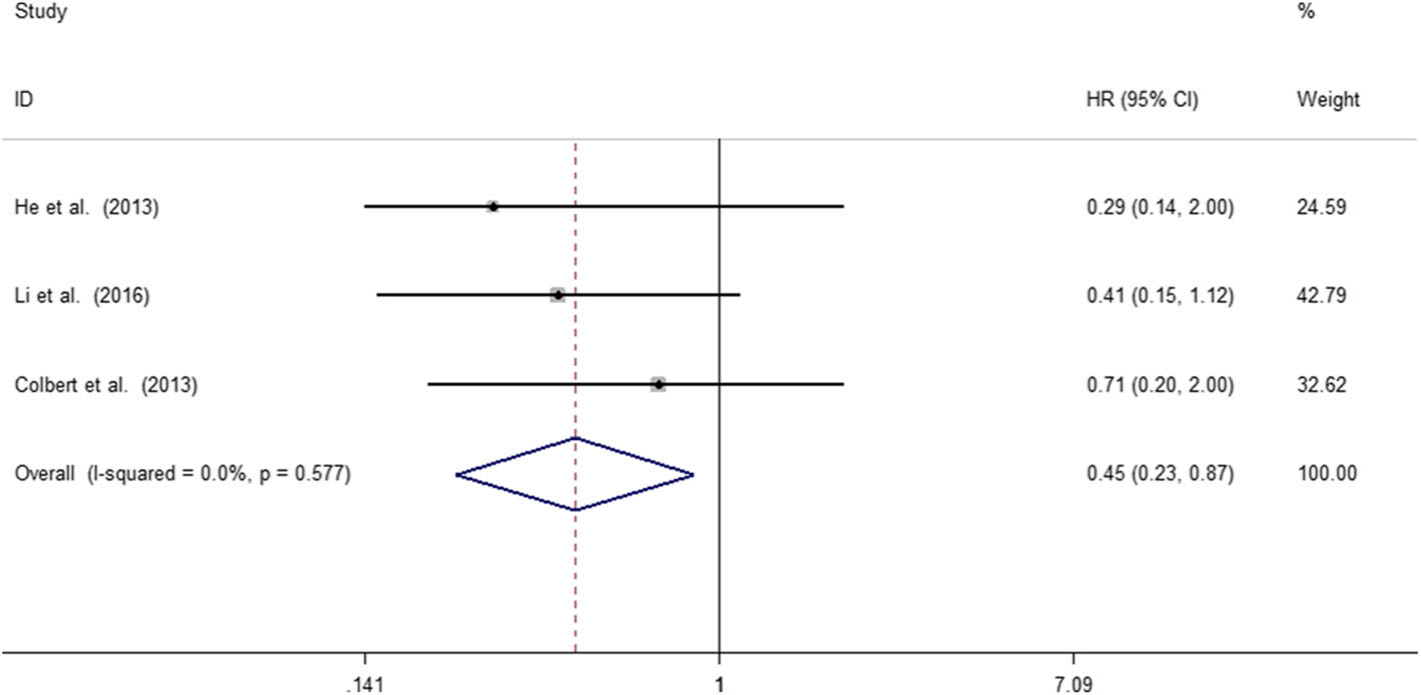
Meta-analysis of the pooled HRs for EFS in cancer patients

A total of three studies including 368 patients reported the impact of abnormally expressed MLKL on DFS or RFS of cancer patients. In the current study, we defined DFS and RFS as EFS. The consequence displayed that decreased expression level of MLKL was significantly associated with poor EFS in cancer patients (HR = 0.45, 95%CI: 0.23–0.87, *p* = 0.017) (Fig. 4). There was no significant heterogeneity among studies (X^2^ = 1.10, *p* = 0.577; I^2^ = 0.0%). However, due to the limited number of included studies, we did not performed the subgroup analysis.

### Association between MLKL expression levels with clinicopathological characteristics of cancer patients

As shown in Table 3, we analyzed the association between MLKL expression levels with clinicopathological characteristics of cancers patients. The meta-analytic results showed that decreased expression level of MLKL was significantly associated with advanced tumor stage (OR = 1.81, 95% CI: 1.09–3.01, *p* = 0.021), more lymph node metastasis (OR = 3.83, 95% CI: 2.29–6.40, *p* < 0.001) and older age (OR = 1.93 95% CI: 1.28–2.93, *p* = 0.002). However, there was no significant association between decreased expression of MLKL with the differentiation grade (OR = 0.86, 95% CI: 0.16–4.57, *p* = 0.857) and gender (OR = 1.41, 95% CI: 0.85–2.36, *p* = 0.188).

**Table 3:** Association between MLKL and clinicopathological characteristics of cancer patients

Clinicopathological parameters	Studies (n)	Patients (n)	OR (95% CI)	*p* value	Heterogeneity		
					I^2^ (%)	p	Model
Differentiation grade (poorly and moderately VS well)	2	252	0.86 (0.16–4.57)	0.857	69.2%	0.071	Random
Gender (male vs. female)	2	252	1.41 (0.85–2.36)	0.188	0%	0.609	Fixed
Lymph node metastasis (yes vs. no)	4	380	3.83 (2.29–6.40)	< 0.001	0%	0.478	Fixed
Tumor stage (III–IV vs. I–II)	3	326	1.81 (1.09–3.01)	0.021	21.7%	0.279	Fixed
Age (> 60vs. ≤ 60 years)	3	406	1.93 (1.28–2.93)	0.002	0%	0.569	Fixed

### Sensitivity analysis and publication bias

**Fig. 5:**
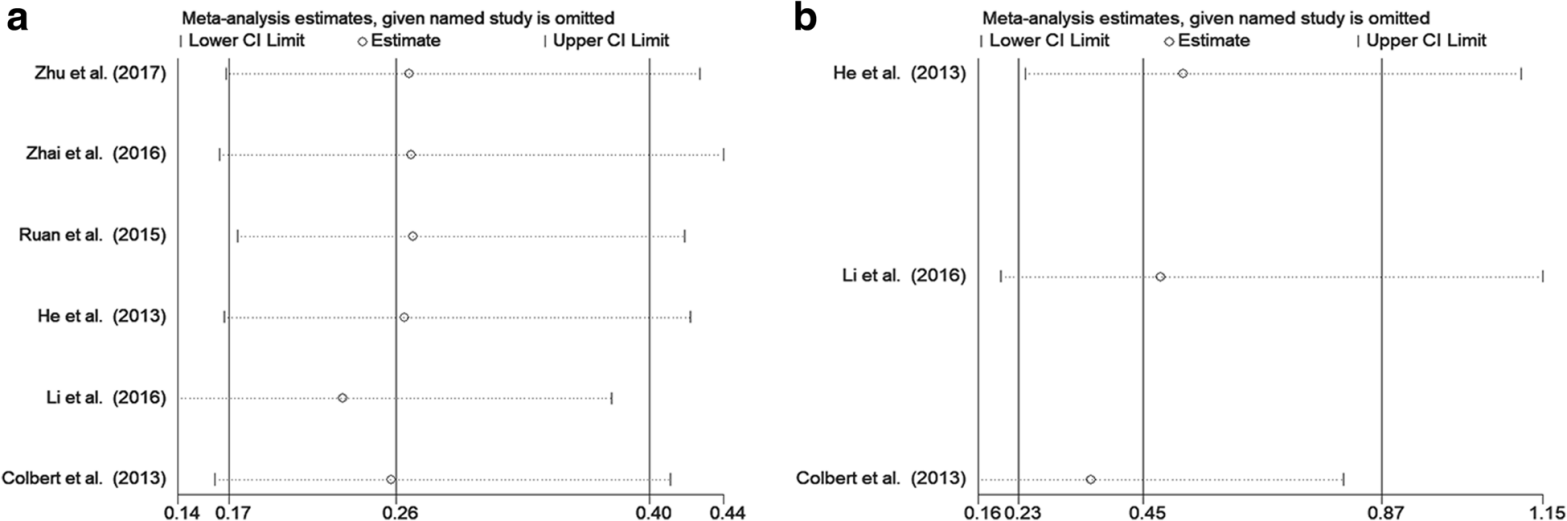
Sensitivity analysis plot of pooled HRs of OS (**a**) and EFS (**b**) for cancer patients with abnormally expressed MLKL

Sensitivity analysis was performed to examine the effects of individual study on the overall results. For OS, the sensitivity analysis identified that result from Li et al. affected pooled HR greatly, indicating that this study was possible to be the main source of heterogeneity. However, after excluding this study, we still observed that decreased expression level of MLKL was significantly associated with poor OS in cancer patients (Fig. 5a). For EFS, sensitivity analysis showed that HRs and their 95% CIs did not change significantly after the exclusion of any of the studies, which indicated that individual study had little influence on our eventual outcome, and proved that our analysis was relatively stable and credible (Fig. 5b). As for publication bias analysis, because of the limited number of studies (below 10) included in each analysis, publication bias was not assessed.

## Discussion

Necroptosis is the most well-studied form of regulated necrosis, which could be triggered by death receptors ligands and stimuli that induce the expression of death receptor ligands under apoptotic deficient conditions [20]. Accumulating evidence has demonstrated that necroptosis plays pivotal roles in the pathogenesis of cancer. MLKL is the most important executor of necroptosis pathway. Decreased expression of MLKL has been found in many kinds of tumors, such as colon cancer, ovarian cancer, cervical squamous cell cancer, pancreatic adenocarcinoma and gastric cancer [21–23, 25, 26]. Recent studies have demonstrated that MLKL could serve as a potential prognostic biomarker for patients with carcinoma. However, most studies reported so far are limited in discrete outcome and sample size.

Here we performed current meta-analysis to explore the prognostic value of abnormally expressed MLKL and the relation between MLKL expression levels with clinicopathological characteristics as well as to further reveal the prognostic value of necroptosis in cancer patients. We examined 6 independent studies comprising data from a total of 613 patients. Through systematic analysis, our results demonstrated that decreased expression level of MLKL was significantly associated with poor OS in cancer patients. In addition, the subgroup analysis results displayed that factors including type of cancer, sample size, follow-up time and paper quality did not alter above results. Furthermore, by combining HRs from studies using Cox multivariate analysis, we found that MLKL was an independent prognostic factor of OS in cancer patients. DFS and RFS are important parameters reflecting the progression of tumor. In this article, we defined DFS and RFS as EFS. The prognostic significance of MLKL in EFS was evaluated in 3 studies with 368 patients. The results indicated that decreased expression level of MLKL was significantly associated with poor EFS of cancer patients.

As for the clinicopathological characteristics, our analysis revealed that decreased expression level of MLKL was significantly associated with advanced tumor stage, more lymph node metastasis and older age. However, there was no significant association between MLKL expression levels with gender or differentiation grade of patients.

Mechanisms underlying the regulatory role of MLKL in tumorigenesis and tumor progression have been extensively investigated. In cancer cells, RIPK3 interacts with and phosphorylates MLKL to promote necroptosis [30]. MLKL is the major executioner of necroptosis downstream of RIPK1/RIPK3. Given the pivotal role of MLKL in necroptosis, one possible underlying mechanism for the association between low MLKL expression levels with poor prognosis of cancer patients may be the result of decreased necroptosis signaling, leading to the resistance to death for cancer cells in these patients. Furthermore, it has been proven that a growing list of compounds, anticancer drugs and several kinase inhibitors could initiate necroptosis in different cancer cells. In addition, radiation and chemotherapy could also induce necroptosis [31]. Thus, patients with low MLKL expression levels in tumor tissues may be less likely to benefit from the regular anti-tumor treatment, which results in the poor prognosis of cancer patients. This is in accordance with the result from Li et al. that in the patients receiving adjuvant chemotherapy, low MLKL expression is associated with decreased RFS and OS.

In our study, a few limitations should be underlined. First, only 6 studies were included in our meta-analysis, and even fewer articles, three articles were included for the EFS analysis, this restricted our ability to evaluate the prognostic value of MLKL in subgroup analysis and might lead to the bias of the results. Second, due to the limited number of included articles, we could not perform the publication bias analysis, which was possible to exist in our meta–analysis. Third, although all studies used IHC to evaluate the expression of MLKL, the cut-off value differed among studies, which might cause bias in the meta-analysis. Fourth, we calculated some HRs through survival curves, it might not be precise enough. Therefore, larger-scale, multicenter and high-quality studies are desperately necessary to confirm our findings.

## Conclusions

In conclusion, our study revealed that decreased expression level of MLKL was significantly associated with poor OS and EFS in cancer patients. Moreover, the expression level of MLKL was associated with clinicopathological features including TNM stage, lymph node metastasis and age. This is the first meta-analysis to evaluate the relationship between expression levels of MLKL, the critical component in necroptosis pathway, with prognosis of cancer patients. In the future, more relevant studies are warranted to investigate the role of MLKL in human cancer.

## Abbreviations

95% CIs: 95% confidence intervals
DFS: Disease-free survival
EFS: Event-free survival
HR: Hazard ratios
MLKL: Mixed lineage kinase domain-like protein
NOS: The Newcastle–Ottawa Quality Assessment Scale
OR: Odds ratios
OS: Overall survival
qRT-PCR: Quantitative real time PCR
RFS: Recurrence -free survival
RIPK1: Receptor-interacting protein kinase 1
RIPK3: Receptor-interacting protein kinase 3
